# Pirtobrutinib for the Treatment of Therapy‐Resistant Chronic Lymphatic Leukemia With CNS Localization

**DOI:** 10.1155/crh/1249723

**Published:** 2026-10-20

**Authors:** M. E. Roerink, A. P. Kater, S. H. Tonino, E. van der Spek

**Affiliations:** ^1^ Department of Hematology Radboud UMC Nijmegen the Netherlands; ^2^ Department of Hematology Amsterdam UMC Amsterdam the Netherlands amc.nl; ^3^ Department of Hematology Rijnstate Ziekenhuis Arnhem the Netherlands

## Abstract

During the last decade, many new treatment options for chronic lymphatic leukemia (CLL) have become available, one of them being covalent‐binding Bruton’s tyrosine kinase inhibitors (cBTK inhibitors [cBTKis]). There are currently three cBTKis approved for treatment of CLL/SLL: acalabrutinib, ibrutinib, and zanubrutinib. In relapsed and heavily pretreated patients, BTK inhibitors achieve a median PFS of 4 years, and in frontline, around 80% of CLL patients are still in remission and alive after 4 years of treatment with BTK inhibitors. Despite their efficacy, 40% of patients have to discontinue BTK inhibitors because of side effects or treatment resistance. We present the efficacy of the novel noncovalent BTKi in a patient with CLL who developed covalent BTKi‐resistant disease with CNS localization. Pirtobrutinib appears to be effective in patients with ibrutinib resistance, even with CNS localization. Clinical responses may take several months to become evident.

## 1. Background

BTK inhibitors are an important treatment modality in patients with chronic lymphatic leukemia (CLL). Ibrutinib was the first approved BTK inhibitor, but the more recently introduced compounds acalabrutinib and zanubrutinib have demonstrated safety advantages compared to ibrutinib in the relapsed/refractory setting. While BTKi improve outcomes for patients with CLL, they are not curative. Despite their efficacy, both side effects and treatment resistance are important reasons for treatment discontinuation in up to 40% of patients [1]. Approximately one‐third of patients who have to discontinue treatment stop because of disease progression, and half of patients as a consequence of toxicity. The most common side effects of BTK inhibitors are hypertension, increased bleeding risk, atrial fibrillation, painful joints, and diarrhea [2].

The mechanism of treatment resistance has not been completely elucidated. Contributing mechanisms include the development of mutations (most commonly, the Cys481 residue within the ATP‐binding site of BTK [3]), limited bioavailability, and the relatively short half‐life. Importantly, the available BTKi share similar resistance mechanisms and therefore are not effective when used in sequence in the setting of drug resistance to another member of the drug class. Consequently, once patients progress on any of these agents, remaining therapeutic options are limited.

To overcome these problems, the novel reversible, noncovalent BTK inhibitor pirtobrutinib (LOXO‐305) has been developed [4]. In comparison with the irreversible, covalent BTK inhibitors described above, pirtobrutinib has a higher BTK selectivity and also appears effective in patients with previous BTK resistance due to Cys481 mutations. We here present our experience with pirtobrutinib by presenting the case of a CLL patient previously treated with several lines of therapy and who was also known to have a central nervous system localization of CLL.

## 2. Case Presentation

A 67‐year‐old patient with CLL has been treated at our clinic since 2006. At presentation, he had splenomegaly, pathological lymph nodes, and a mild thrombopenia. Previously, the patient had been diagnosed with recurrent venous thrombosis warranting the use of anticoagulant drugs. In 2012, he had a severe pneumococcal sepsis as a consequence of an earlier splenectomy. It was complicated by necrosis of his extremities that resulted in bilateral amputation of his lower legs and arms. At diagnosis, the patient had an unmutated IgVH status (VH3‐21DJ) and no cytogenetic abnormalities, but in 2013, after FCR, a 17p deletion was found. In 2017, he presented with a CNS localization manifesting as loss of strength in his upper legs, which was confirmed by the presence of CLL in the cerebrospinal fluid (CSF). He was treated with venetoclax intravenously with a partial response and return of strength up until progression. The use of a vitamin K antagonist was a contraindication for starting a BTK inhibitor. Three years later, in January 2020, our patient again developed neurological symptoms, which mainly existed due to loss of strength. He then switched to ibrutinib (420 mg), with again a clinical partial response. During this treatment, our patient developed hypertension, which was treated effectively with blood pressure‐lowering drugs. After 1.5 years, lymphocytes and lactate dehydrogenase (LDH) increased rapidly, and the patient presented with bleeding of the oral mucosa and bruising of the skin. Eventually, ibrutinib was stopped, and pirtobrutinib was started at a dose of 200 mg a day during 28‐day cycles in December 2021 (Eli Lilly and Company, Patient UseIlly compassionate use program). Before starting pirtobrutinib at the end of 2021, six different treatment lines had been given (Table 1). At this moment, he developed a BTK C481S mutation, as well as a TP53 mutation with a VAF of 58%. There was no PCLG2 mutation.

**TABLE 1:** Treatment overview before start of pirtobrutinib.

Date	Treatment	Reason for discontinuation
2007	Chlorambucil	Thrombopenia
2008‐02	Fludarabine–cyclophosphamide	Complete remission
2010‐06	Rituximab–fludarabine–cyclophosphamide	Completed 3 cycles
2013‐09	PI3K inhibitor (clinical trial)	End of study (2016‐4)
2016‐05	Idelalisib (PI3K inhibitor)	CNS localization
2017‐02	Venetoclax	Recurrent CNS localization
2020‐1	Ibrutinib	Recurrent disease
2021‐12	Pirtobrutinib	Progressive disease (23‐5)

Table 2 displays laboratory results before the start of the treatment and during the months thereafter.

**TABLE 2:** Laboratory results before and during treatment with pirtobrutinib.

Date	7/27/21	10/6/21	12/21/21	1/17/22	1/26/22	3/14/22	6/10/22	9/5/22
Lymphocytes (× 10^9^/L)	**8.1**	**45.5**	**166.8**	**217.2**	**223.5**	**158.9**	**80.6**	**21.1**
LD (U/L) (< 250)	**298**	**586**	**1103**	**279**	**310**	**242**	**237**	**201**

*Note:* Bold values indicate lymphocyte and ldh levels.

In the first weeks after starting treatment with pirtobrutinib, there appeared to be a good response with a decrease in fatigue, disappearance of bleeding symptoms, and a significant decrease in LDH. The lymphocytes further increased, but the LDH started to normalize. However, at 1 month of treatment, the patient complained of weakness in his legs again and problems in controlling defecation, symptoms he recognized from earlier when CNS localization of CLL was diagnosed. An MRI scan supported our suspicion of CLL localization in the cauda equina, as reflected by thickening without evidence of a discrete mass. CSF analysis revealed monoclonal lymphocytosis, phenotypically compatible with low‐grade CLL localization. Treatment with pirtobrutinib was continued, and intrathecal methotrexate twice a week was initiated. After 2 months from the start of pirtobrutinib and 1 month from the start of methotrexate biweekly, the patient requested discontinuation of intrathecal methotrexate because of the difficulties experienced with administration in the absence of any clinical improvement. This means treatment with methotrexate was only given for four administrations.

In the 3rd month, the lymphocytes started to decline, although the patient did not notice any improvement in his neurological situation. In the 4th month, the patient was able to stand and experienced an improvement in his condition. Five months from the start of pirtobrutinib, the patient was able to walk again (with his prosthetic legs), showing ongoing improvement in his laboratory results. Unfortunately, 16 months from the start of pirtobrutinib, the patient experienced new progression of his CLL and developed fatigue and malaise, and he chose palliative care. He died after 2 weeks at home.

## 3. Discussion

This patient with CLL confirms the results of the BRUIN trial showing efficacy in an extensively treated patient. We describe a case of a patient with CLL who had an excellent treatment response after starting the noncovalent BTK inhibitor pirtobrutinib without the occurrence of side effects. Right after the start of treatment, there was an increase in the number of leukocytes, which is a known phenomenon after the start of a BTK inhibitor. It is assumed that this increase is a result of lymphocytes leaving tissues and entering the circulation [5]. In our patient, pirtobrutinib appears to be effective despite earlier disease progression during treatment with ibrutinib, a covalent BTK inhibitor, and in the presence of a proven BTK C481S mutation.

While pirtobrutinib is approved for use in relapsed/refractory CLL following prior BTKi therapy, its role in CNS involvement remains to be defined. In the previously mentioned “BRUIN” study [6], 86% of the included patients had previously been treated with covalent‐binding Bruton’s tyrosine kinaseinhibitors (cBTKis) and had to discontinue as a result of toxicity (33%) or CLL progression (67%). A median of three other treatment lines had preceded the current treatment. The treatment effect of pirtobrutinib could be evaluated in 139 patients with CLL. There was an overall response rate of 63%, and 62% in patients previously treated with a cBTKi. During the follow‐up period, five patients discontinued treatment, 1 patient had disease progression, and the remaining group of patients had stable disease or an improvement. With respect to Grade 3/4 toxicity, the most frequently reported side effect was neutropenia in 5% of patients. The usual side effects of BTK inhibitors, such as hypertension, bleeding, and atrial fibrillation, were not reported in this group. Comparable results were achieved in the follow‐up Phase III study [7].

Although our patient initially had disease progression after the start of treatment with development of CNS localization, after 3 months, there was a treatment response with disappearance of neurological symptoms. Methotrexate was only given for 4 weeks, and we do not believe it has contributed to the improvement months later. CNS localization is a rare phenomenon in CLL, although in postmortem studies, it can be found in 20% of patients [8]. The CNS effect of the noncovalent BTKi has not been extensively studied yet, but there is a known effect of the cBTKi in patients with CNS localization. For example, zanubrutinib has been proven to be effective in primary CNS lymphoma [9, 10]. However, the effect of pirtobrutinib might take time, with initial worsening of symptoms in our patient. The reason for this remains to be elucidated but might be caused by partial CNS penetration.

Studies evaluating the effect of pirtobrutinib are ongoing to better define the added value of noncovalent BTKi. Considering the poor prognosis of patients not responding to cBTKis, pirtobrutinib might give this patient group some perspective, even in the case of CNS localization.

## 4. Take Home Messages

1. Because of treatment resistance and side effects, almost half of patients have to discontinue the classic, covalent BTK inhibitors.
2. Pirtobrutinib is one of the newer reversible noncovalent BTK inhibitors and is also effective in patients previously treated with ibrutinib.
3. Pirtobrutinib is also effective in CLL patients with CNS localization.

## Funding

No funding was received for this study.

## Disclosure

All authors have read and approved the final version of the manuscript. Roerink M. E. had full access to all of the data in this study and takes complete responsibility for the integrity of the data and the accuracy of the data analysis.

## Conflicts of Interest

The authors declare no conflicts of interest.

## Supporting Information

Additional supporting information can be found online in the Supporting Information section.

## Supporting information


**Supporting Information** Care checklist.

## Data Availability

Data sharing is not applicable to this article as no datasets were generated or analyzed during the current study.
